# Inulin impacts tumorigenesis promotion by colibactin-producing *Escherichia coli* in *Apc^Min/+^* mice

**DOI:** 10.3389/fmicb.2023.1067505

**Published:** 2023-02-02

**Authors:** Manon Oliero, Roy Hajjar, Thibault Cuisiniere, Gabriela Fragoso, Annie Calvé, Manuela M. Santos

**Affiliations:** ^1^Nutrition and Microbiome Laboratory, Institut du cancer de Montréal, Centre de recherche du Centre hospitalier de l’Université de Montréal (CRCHUM), Montréal, QC, Canada; ^2^Department of Surgery, Faculty of Medicine, Université de Montréal, Montréal, QC, Canada; ^3^Department of Medicine, Faculty of Medicine, Université de Montréal, Montréal, QC, Canada

**Keywords:** inulin, *Apc^Min/+^* mouse, colibactin, tumorigenesis, double-strand DNA breaks, *pks+ E. coli, E. coli Nissle*

## Abstract

**Introduction:**

The prebiotic inulin has previously shown both protective and tumor-promoting effects in colorectal cancer (CRC). These inconsistencies may be due to the gut microbial composition as several bacteria have been associated with CRC. Specifically, polyketide synthase-positive (*pks+*) *Escherichia coli* promotes carcinogenesis and facilitates CRC progression through the production of colibactin, a genotoxin that induces double-strand DNA breaks (DSBs). We investigated whether colibactin-producing *Escherichia coli* changed the protection conferred by inulin against tumor growth and progression using the *Apc^Min/+^* mouse model of CRC.

**Methods:**

Mice received a 2% dextran sodium sulfate (DSS) solution followed by oral gavage with the murine *pks + E. coli* strain NC101 (EcNC101) and were fed a diet supplemented with 10% cellulose as control or 10% inulin for 4 weeks.

**Results:**

Inulin supplementation led to increase EcNC101 colonization compared to mice receiving the control diet. The increased colonization of EcNC101 resulted in more DSBs, tumor burden, and tumor progression in *Apc^Min/+^* mice. The tumorigenic effect of EcN101 in *Apc^Min/+^* mice mediated by inulin was dependent on colibactin production. Pasteurized *E. coli* Nissle 1917 (EcN), a probiotic, suppressed the inulin-driven EcNC101 expansion and impacted tumor progression.

**Discussion:**

Our results suggest that the presence of *pks + E. coli* influences the outcome of inulin supplementation in CRC and that microbiota-targeted interventions may mitigate this effect. Given the prevalence of *pks + E. coli* in both healthy and CRC populations and the importance of a fiber-rich diet, inulin supplementation in individuals colonized with *pks +* bacteria should be considered with caution.

## 1. Introduction

Colorectal cancer (CRC) is the third most diagnosed cancer worldwide (Xi and Xu, 2021). The composition and function of the gut microbiome have been shown to potentially play a major role in the initiation and progression of CRC (Song et al., 2020). Patients with CRC have an imbalanced gut microbiome or dysbiosis, reflecting a decrease in beneficial bacteria and an increase in pathobionts, such as polyketide synthase positive (*pks+*) *Escherichia coli* (Wirbel et al., 2019). The *pks* genomic island includes the colibactin (*clb*) gene cluster, which encodes the genes necessary for the production of colibactin (Homburg et al., 2007), a genotoxin that induces double-strand DNA breaks (DSBs), cell cycle arrest, senescence, and chromosomal abnormalities in mammalian cells, contributing to a CRC-specific mutational profile (Pleguezuelos-Manzano et al., 2020). Murine models of *pks + Escherichia coli* mono-colonization (Arthur et al., 2012; Dejea et al., 2018) and colonization of *Apc^Min/+^* mice with colibactin producing *E. coli* (Lopès et al., 2020) revealed a causal link between the presence of colibactin and intestinal tumorigenicity. Colonization with colibactin-producing *E. coli* in humans occurs early in life (Tsunematsu et al., 2021) and is steadily increasing worldwide (Fais et al., 2018). Up to 68% of CRC patients are found colonized with *pks + E. coli* (Eklof et al., 2017; Shimpoh et al., 2017; Dejea et al., 2018; Iyadorai et al., 2020; Nouri et al., 2021).

Given the causal link between *pks + E. coli* and sporadic colon cancer development (Pleguezuelos-Manzano et al., 2020), dietary recommendations and microbiota-targeted therapies have emerged. Dietary fibers and prebiotics may play a crucial role in regulating the gut microbiome (Gibson et al., 2017) and CRC prevention (Mahdavi et al., 2021). For instance, the prebiotic inulin may benefit gut health through its ability to maintain the integrity of the protective mucus layer while increasing the amount of beneficial and commensal bacteria (Pham et al., 2018), which provide a protective barrier against opportunistic colonization and excessive growth of pathobionts, such as *E. coli* (Fooks and Gibson, 2002). However, clinical data on the effect of inulin on cancer development remain inconsistent due to an absence of evidence showing a direct positive impact on tumorigenicity (Mazraeh et al., 2019). Limburg et al. demonstrated that inulin supplementation did not reduce aberrant crypt foci (ACF) (Limburg et al., 2011), and we have previously reported that *in vitro*, inulin may promote the genotoxicity of colibactin-producing bacteria on mammalian cells (Oliero et al., 2021).

In this study, we aimed to evaluate whether the presence of *pks + E. coli* influenced the outcome of inulin supplementation in a CRC context using the *Apc^Min/+^* mouse model. Mice were fed a diet supplemented with cellulose or inulin and colonized with the murine colibactin-producing *E. coli* strain NC101 (EcNC101) to explore the role of inulin on EcNC101 in intestinal tumor progression.

## 2. Materials and methods

### 2.1. Bacterial strains

*E. coli* strains in this study included the pathogenic murine strain EcNC101 wild-type (WT), the EcNC101 depleted for the *colibactin P* gene (*ΔclbP*) (both EcNC101 strains were a gift from Dr. Christian Jobin, Cancer Microbiota & Host Response, UF Health Cancer Center, University of Florida), the probiotic *E. coli* Nissle 1917 (EcN) (Mutaflor®, Pharma-Zentrale GmbH, Germany) and the *E. coli* K12 (EcK12) (ER2738, New England BioLabs Ltd., Whitby, ON, Canada). EcNC101 were transformed with the plasmid pROP-IP-FBFP (a gift from Srivatsan Raman; Addgene plasmid #122138^1^; RRID: Addgene_122,138) that encodes a flavin mononucleotide-based fluorescent protein (FbFP) to obtain EcNC101-FbFP. All strains of *E. coli* were grown from glycerol stocks in lysogeny broth (LB) with adequate antibiotic supplementation at 37°C at 150 revolution per minute (rpm) overnight and subsequently subculture in appropriate media. The *Lactobacillus plantarum* 299 V® (Digestive care, Jamieson Wellness Inc., Toronto, ON, Canada) was grown overnight at 37°C in Man-Rogosa-Sharpe (MRS) broth (Sigma–Aldrich Canada Co, Oakville, ON, Canada) in anaerobic conditions.

### 2.2. Animal experiments

Following the Canadian Council of Animal Care guidelines, all procedures were performed after approval by the Institutional Animal Care Committee of the Centre de recherche du Centre Hospitalier de l’Université de Montréal (CRCHUM). Breeding colonies of WT and *Apc^Min/+^* C57BL/6 mice (Jackson Laboratory, Bar Harbor, ME, United States) were established in a specific-pathogen-free (SPF) facility, and offspring were genotyped using allele-specific polymerase chain reaction (PCR) analysis. Mice were 4 weeks old at the beginning of the experiments and were maintained under standard 12:12 light/dark conditions. They were co-housed at two to three mice per cage and were allowed *ad libitum* access to food and water. Mice received a standard diet (Envigo Teklad Diets, TD2918) until 5 weeks old. At 4 weeks old, *Apc^Min/+^* females were treated with 2% (w/v) dextran sulfate sodium (DSS) (TDB consultancy AB, Uppsala, Sweden) in drinking water for a week. The following day after DSS treatment, mice were fed a diet containing 10% (wt/wt) inulin (Quadra Chemicals Ltd., Vaudreuil-Dorion, QC, Canada) (Envigo Teklad Diets, TD.190651) or a control diet containing 10% (wt/wt) cellulose (Envigo Teklad Diets, TD.190723), a non-fermentable fiber. Apart from fiber supplementation, both diets were matched in caloric intake in terms of carbohydrate, protein, and fat (Supplementary Table S2). At the same time, mice received an oral gavage of 200 μL of bacterial suspension (10^8^ colony-forming units, CFUs) or sterile Dulbecco’s phosphate-buffered saline (D-PBS; WISENT Inc., St-Bruno, QC, Canada) as control. For the competition experiment, mice received an oral gavage of 200 μL of pasteurized EcN (10^8^ CFU) weekly for 3 weeks. At 9 weeks of age, mice were anesthetized with an intraperitoneal injection of sodium pentobarbital and mechanically killed by cervical dislocation. The small intestine and colon were cut in the longitudinal part, and two persons, blinded to the groups, separately counted the number of tumors. Colonic “Swiss rolls” were fixed by 10% buffered formalin (ChapTec Inc., Montreal, QC, Canada) and then embedded in a paraffin block.

### 2.3. DNA extraction and polymerase chain reaction

Total DNA was extracted from fecal samples from mice with the PowerSoil® DNA Extraction Kit (Qiagen Inc., Toronto, ON, Canada) and PCR was performed using PowerUp™ SYBR™ Green Master Mix (Thermo Fisher Scientific, Waltham, MA, United States) in the RG 3000A R PCR machine (Qiagen Inc.) (Fillebeen et al., 2015). Simultaneous amplification of *colibactin A* gene (*clbA*) and *E. coli* 16 s rRNA were done with the pair of primers clbA and Ecol16S, respectively (Supplementary Table S1). Amplification of *colibactin P* gene (*clbP*) and 16S rRNA were performed using the primers clbP and universal 16S, respectively (Supplementary Table S1). To verify the depletion of the *clbP* gene in the EcNC101 mutant, we used three primers CheckClbP (Supplementary Table S1).

### 2.4. Fluorescence imaging

Colon “Swiss rolls” were placed in optimal cutting temperature (OCT) compound and frozen on dry ice. Sections cut at 5-μm thickness were fixed with 4% paraformaldehyde, permeabilized in 0.1% triton in phosphate-buffered saline (PBS) and blocked in PBS containing 1% bovine serum albumin (BSA) and 4% serum. DNA was stained with DAPI, followed by F-actin staining with Alexa Fluor™ 647 Phalloidin (1/200 in methanol 1X) (a gift from Dr. Larochelle, CRCHUM, University of Montreal). Mucus layer was imaged using acid-Schiff-Alcian blue (Artisan Alcian Blue kit, AR160, Dako North America Inc. Carpinteria, CA; PAS, Sigma–Aldrich). All sections were scanned using a *VS*-110 microscope with a 40× 0.75 NA objective and a resolution of 0.3225 mm (Olympus), and images were then generated using Fiji software.

### 2.5. Histological scoring of tumor grade

Sections were cut at 4-μm thickness and stained with hematoxylin and eosin (H&E), and assessed for low grade adenomas, high grade adenomas, and intramucosal carcinomas.

### 2.6. Immunohistochemistry

Formalin-fixed paraffin-embedded (FFPE) sections of colonic tissue were stained using the Benchmark XT autostainer (Ventana Medical Systems, Tucson, AZ, United States). Immunohistochemical staining was carried out on frozen sections using specific antibodies anti-Ki-67 (Biocare CRM325A, Biocare Medical, Pacheco, CA, United States) and the anti-phospho-γ-H2AX (Ser139, Cell Signaling Technology #9718, New England BioLabs Ltd.). Reactions were performed using the iView DAB detection kit, and counterstaining was achieved with hematoxylin and bluing reagents at 1/150 dilution. DSBs and proliferative indexes were represented by the number of positive cells in the descending colon.

### 2.7. *Pks + Escherichia coli* quantification

Samples of digesta (small intestine) and feces (colon) (Wienk et al., 1997) were plated on MacConkey agar (Thermo scientific Oxoid, Nepean, ON, Canada) and *E. coli* was quantified based on the number of red colonies (small intestine: CFUs/mL; colon: CFUs/g).

### 2.8. Inulin fermentation

*Lactobacillus plantarum* 299 V® was used as a positive control for inulin fermentation and identified by PCR using the pair of primers Plantarum (Supplementary Table S1). Bacteria were sub-cultured at 1/100 dilution in standard minimal medium (M9) supplemented with 1% inulin for 24 h in anaerobic conditions using an anaerobic sachet (BD BBL™ GasPak™ anaerobic indicator, BD, Mississauga, ON, Canada) and the optical density at 600 nm (OD600) was determined in a Spark® multimode microplate reader (Tecan Group Ltd., Männedrof, Switzerland) every hour for a day. In parallel, the release of free fructose and glucose in the supernatants during inulin fermentation were determined after 3 h of culture using a D-fructose/D-glucose assay kit (Megazyme International Ltd., Wicklow, Ireland).

### 2.9. Bacterial competition

To measure EcNC101 growth compared to EcN growth, we transformed strains with the plasmid pUCP20T-E2Crimson and pUCP20T-morange (gifts from Mariette Barbier; Addgene plasmid #78473^2^; RRID:Addgene_78,473/Addgene plasmid #78468^3^; RRID:Addgene_78,468) to distinguish them by crimson and orange fluorescence (Barbier and Damron, 2016). Electroporation was used to insert plasmids into the bacteria. Beforehand, we measured the effect of the plasmid on the growth of the bacteria and validated the fluorescence intensity by transfecting each strain with each plasmid and compared their growth to a control strain without plasmid. There were no variations in the bacteria’s growth or fluorescence intensity. Next, the competitor strains (10^7^CFUs/mL) were inoculated into a starting volume of 5 ml LB and grown at 37°C for 24 h without shaking. After 1 day, 50 μL of the competitor-strains culture was inoculated into fresh LB (1/100) and grown for 24 h, and this was repeated for 3 days. Fluorescence was recorded every day from the starting point in a Spark® multimode microplate reader (Tecan Group Ltd). To measure the effect of pasteurized EcN on EcNC101 growth, EcN was inoculated in fresh LB medium overnight at 37°C. Then, the suspension was centrifuged at 5,000 rpm for 3 min. Finally, the cell pellet was resuspended in fresh LB medium and pasteurized at 70°C for 30 min. EcNC101 was inoculated at 10^7^ CFUs/mL in LB medium composed of 50% or 100% of pasteurized EcN. OD600 was measured at 0, 3, 6, and 24 h.

### 2.10. Statistics

All data were analyzed using GraphPad Prism (Version 5.0, GraphPad Software, San Diego, CA, United States). *χ*^2^ tests were used to compare categorical variables. When the data did not pass the normality test, log(Y) transformation was applied to the data. An analysis of variance (ANOVA) was followed by *post hoc* Tukey’s multiple comparisons test. For tumor count, non-parametric Mann–Whitney test was used. *p*-values <0.05 were considered statistically significant.

## 3. Results

### 3.1. Inulin promotes tumor development in *Apc^Min/+^* mice colonized with EcNC101

**Figure 1:**
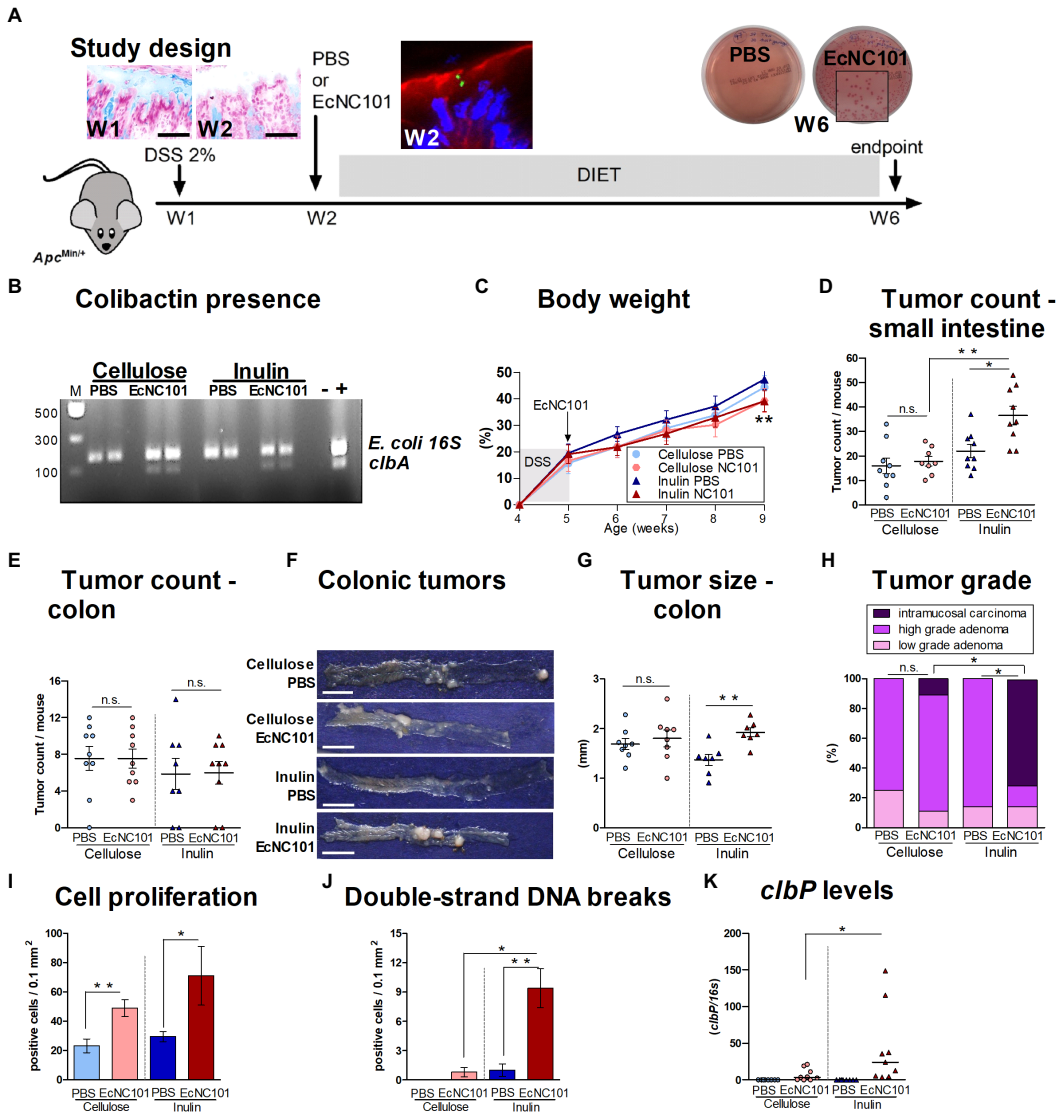
Carcinogenicity in *Apc^Min/+^* mice colonized with EcNC101 and fed an inulin supplemented diet. **(A)** Alcian blue staining of the colonic mucus layer in *Apc^Min/+^* mice after treatment with 2% DSS in drinking water for a week (Week 1, W1). Mice were fed diets supplemented with 10% cellulose or 10% inulin and received oral gavage with PBS or 10^8^ CFU of EcNC101 at W2. EcNC101 FbFP fluorescence observed 24 h after the gavage by confocal microscopy (F-actin: red, DAPI: blue). At the end of the experiment (W6), fecal homogenates were cultured on MacConkey agar for *Enterobacteriaceae* detection. **(B)** EcNC101 colonization assessed by PCR amplification of the *clbA* gene. **(C)** Body weight (mean ± SEM; **p < 0.01 between the inulin groups, repeated-measure ANOVA). **(D,E)** Tumor count in **(D)** small intestine and **(E)** colon. **(F)** Representative images of colonic tumors (left side: cecum, right side: rectum), scale bar = 1 cm. **(G)** Tumor size in the colon. **(H)** Colonic tumor grades (Chi-square Test). **(I)** Quantification of Ki-67-positive cells per healthy mucosa site (*N* = 5 per group). **(J)** Quantification of γ-H2AX-positive cells per healthy colonic mucosa site (*N* = 5 per group). **(K)** *colibactin P* gene by qPCR. n.s. non-significant; *p < 0.05, **p < 0.01. ANOVA in between groups; N = 8–9 per group.

Inulin is a common functional ingredient and supplement with prebiotic benefits (Christoforou et al., 2021). However, our previous findings showed that *in vitro*, inulin may enhance the genotoxicity of *pks + E. coli* that produce colibactin (Oliero et al., 2021). To determine the potential risk of inulin supplementation for CRC development, we investigated whether early colonization with *pks + E. coli* affected the response to inulin supplementation on tumor development in *Apc^Min/+^* mice. As shown in Figure 1A, DSS treatment resulted in temporary damage to the mucus layer, which would promote the direct contact between *pks + E. coli* and intestinal epithelial cells necessary for colibactin action (Nougayrede et al., 2006). Mice received an oral gavage of PBS control or the murine strain of colibactin-producing EcNC101. While all mice had *E. coli* in their gut, colibactin-producing bacteria was only present in mice inoculated with EcNC101 (Figure 1B).

Body weight progress was similar in all four experimental groups during the first 4 weeks, after which, mice colonized with EcNC101 on the inulin diet had a significantly lower body weight compared to the uninfected mice fed the same diet (Figure 1C). As shown in Figure 1D, the number of tumors in the small intestine was significantly higher in *Apc^Min/+^* mice colonized with EcNC101 and fed inulin than in uninfected mice on the same diet (1.7-fold higher). However, EcNC101 colonization did not affect the number of intestinal tumors when mice received the cellulose diet. Finally, when comparing the effect of diet on mice colonized with EcNC101, mice fed the inulin diet had a higher tumor count compared with those fed cellulose (1.7-fold change). These results indicate that the tumorigenic effect of EcNC101 in *Apc^Min/+^* mice in the small intestine was dependent on the diet.

In the colon, the number of tumors was similar in the four groups (Figure 1E). However, the size of the tumors found in EcNC101 positive *Apc^Min/+^* mice fed inulin was significantly larger compared to EcNC101 negative mice on the same diet (1.92 ± 0.15 mm vs. 1.37 ± 0.13 mm, ±SEM; Figures 1F,G). In addition, EcNC101 colonization was associated with a significantly higher tumor grade based on the appearance of intramucosal carcinomas that were never observed in EcNC101 negative mice. In addition, 71% of mice fed the inulin diet had intramucosal carcinomas compared to 11% of mice fed cellulose (Figure 1H; Supplementary Figure 1), indicating that tumor progression in the colon of mice colonized with *pks +* bacteria depends on the diet.

In line with these findings, EcNC101 colonization led to increased cell proliferation as assessed by Ki-67 expression (Luo et al., 2019), regardless of diet, and to increased levels of γ-H2AX staining, a marker of DSBs (Mah et al., 2010), in mice fed the inulin diet (Figures 1I,J; Supplementary Figures 2A,B). Finally, higher levels of the *clbP* gene were present in fecal samples from *Apc^Min/+^* mice fed the inulin diet (Figure 1K).

Overall, these results show that in the presence of colibactin-producing *E. coli*, inulin accelerates tumor progression in the gastrointestinal tract of *Apc^Min/+^* mice.

### 3.2. Higher tumorigenicity in EcNC101-colonized *Apc^Min/+^* mice fed inulin is dependent on colibactin

**Figure 2:**
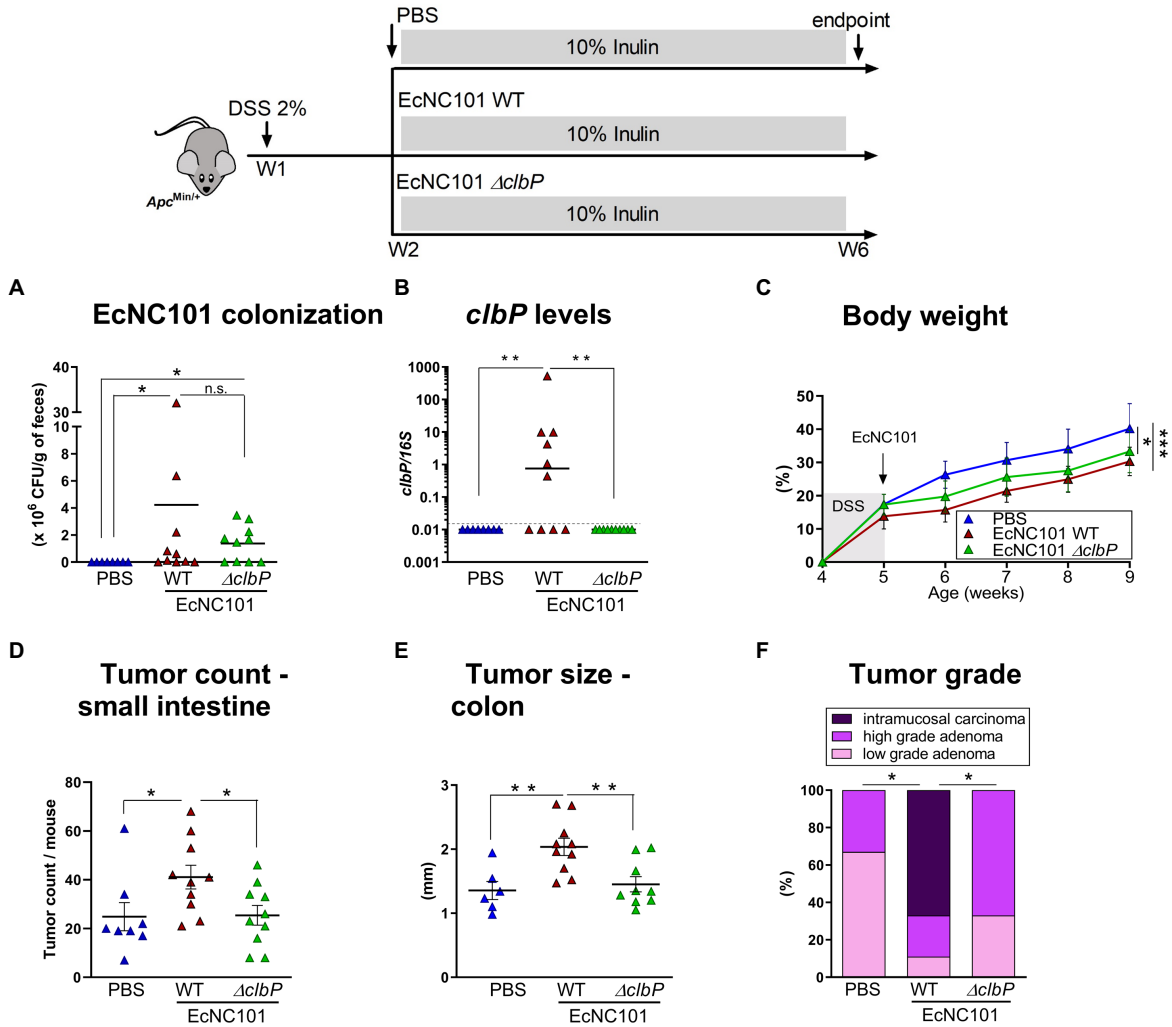
Tumorigenesis in *Apc^Min/+^* mice fed inulin and colonized with wild-type and colibactin-deficient EcN101 strains. **(A)** EcNC101 colonization assessed by CFUs and **(B)** *colibactin P* gene by qPCR in fecal samples. **(C)** Body weight (mean ± SEM; repeated-measure ANOVA). **(D)** Tumor count in the small intestine. **(E)** Tumor size in the colon. **(F)** Colonic tumor grades (Chi-square Test). n.s. non-significant; *p < 0.05, **p < 0.01. ANOVA, N = 8–11 per group.

To investigate whether colibactin is necessary for the enhanced tumorigenesis in mice colonized with *pks + E. coli*, *Apc^Min/+^* mice fed an inulin diet were inoculated with EcNC101 WT or with the colibactin-deficient mutant strain EcNC101 *ΔclbP* (Dubois et al., 2011). The quantification of CFUs revealed similar abundance levels between EcNC101 WT and EcNC101 *ΔclbP* in the feces 4 weeks post-inoculation (Figures 2A,B) indicating that further effects were not caused by colonization levels of the various mutants.

*Apc^Min/+^* mice colonized with WT or *ΔclbP* mutant had a significantly lower body weight compared to the uninfected mice (Figure 2C). However, tumor counts in the small intestine in mice colonized with the *ΔclbP* mutant was comparable to uninfected mice (25 ± 4.0 vs. 25 ± 5.8 tumors; ±SEM) and was significantly reduced compared to mice colonized with the WT strain (41 ± 4.9 tumors; ±SEM Figure 2D). In addition, tumor progression was also reduced in mice colonized with the *ΔclbP* mutant compared to WT as evidenced by tumors of smaller size (1.45 ± 0.11 mm vs. 2.04 ± 0.13 mm; ±SEM) and of lower grade in the colon (Figures 2E,F). Body weight changes in both *WT* and *ΔclbP* EcNC101 positive mice may be due to altered metabolic processes since chronic colonization with pathogenic *E. coli* strains can induce changes in inflammatory-associated genes, such as adiponectin, leptin and thrombopoietin (Stromberg et al., 2018). Alternatively, or concomitantly, *E. coli* colonization may lead to significant changes in gut microbiota composition, known to affect body weight (Million et al., 2013), which are independent of colibactin expression (Tronnet et al., 2020).

These results indicate that the deleterious effect of inulin in *Apc^Min/+^* mice colonized with EcNC101 is dependent on the ability of the *E. coli* strain to produce colibactin.

### 3.3. Inulin enhances EcNC101 growth

**Figure 3:**
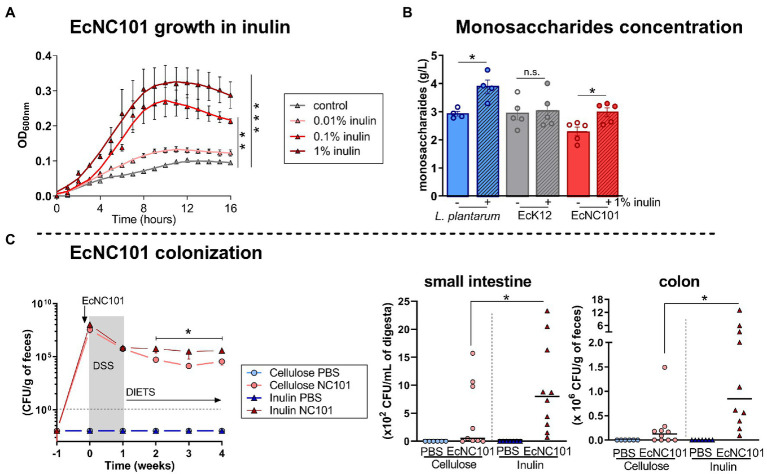
Inulin effect on EcNC101 growth and colonization. **(A)** Growth curves (repeated-ANOVA, compared to control). **(B)** Monosaccharide concentration in the medium inoculated with inulin-fermenting *L. plantarum* (positive control), EcK12 (negative control), and EcNC101 (Student’s *t*-test); **(C)** Fecal homogenates were cultured on MacConkey agar for *Enterobacteriaceae* detection at W 0, 1, 2, 3 and 4. Quantification of EcNC101 colonization by CFUs in the small intestine and colon. n.s. non-significant, *p < 0.05, **p < 0.01, ***p < 0.001. ANOVA.

To further assess the effect of inulin on EcNC101 growth, we cultured the bacteria in the presence of inulin. The addition of inulin increased the growth of EcNC101 in a concentration-dependant manner (Figure 3A). We then investigated whether EcNC101 was able to ferment inulin for their growth and measured the concentration of monosaccharides released in the minimal culture medium inoculated with EcNC101. The inulin-fermenting strain *Lactobacillus plantarum* was used as a control and as expected (Nordstrom et al., 2021), increased the concentration of monosaccharides in the medium (1.4-fold change) with the addition of 1% inulin (Figure 3B). EcK12, used as a control strain unable to ferment inulin (Rossi et al., 2005; Jung et al., 2015), did not change the monosaccharide concentration. In contrast, EcNC101 cultures released monosaccharides into medium when supplemented with 1% inulin (1.4-fold change; Figure 3B). These data suggest that EcNC101 ferments inulin and thus, may explain the effect of inulin in promoting EcNC101 growth.

Next, we quantified EcNC101 colonization in *Apc^Min/+^* mice overtime by culturing fecal homogenates on MacConkey agar plates and counting CFUs. The EcNC101 colonization level were similar until week 2, after which EcNC101 levels in fecal samples were higher in the *Apc^Min/+^* mice fed an inulin supplemented diet compared to those fed the cellulose diet (Figure 3C). CFUs quantification at the endpoint confirmed that EcNC101 was present at higher concentrations in the small intestine and in fecal samples of *Apc^Min/+^* mice fed the inulin diet (ranging from 10^5^ to 10^7^ CFUs/mg feces) compared to mice fed the cellulose diet (ranging from10^3^ to 10^6^ CFUs/mg feces) (Figure 3C).

Together, these results suggest that EcNC101 may ferment inulin to fuel its growth, resulting in increased expansion of EcNC101 in *Apc^Min/+^* mice fed an inulin supplemented diet.

### 3.4. Pasteurized probiotic *Escherichia coli* Nissle inhibits tumorigenic effect of EcN101 in *Apc^Min/+^* mice fed an inulin diet

**Figure 4:**
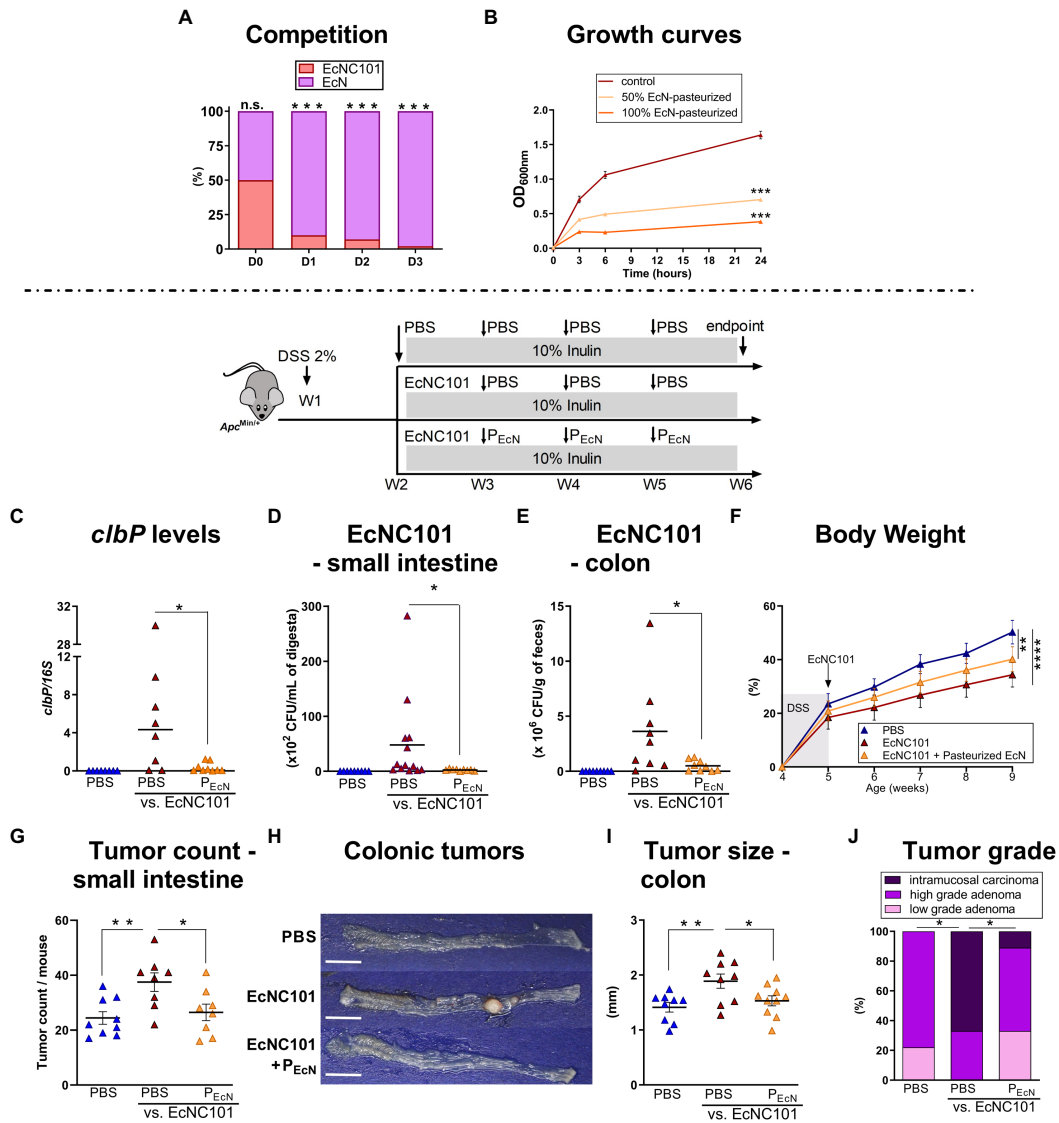
Effect of pasteurized *E. coli* Nissle on EcNC101 growth, tumorigenesis and tumor development in inulin-fed *Apc^Min/+^* mice. **(A)** Competition assay between EcNC101 and EcN for three days (Fisher’s Test). **(B)** OD_600_ of EcNC101 grown in LB supplemented with 50 and 100% pasteurized EcN (repeated-measure ANOVA). **(C–I)** Mice were fed a diet supplemented with 10% inulin and were gavaged with PBS or 10^8^ CFU of EcNC101 at W2, followed by an oral gavage with PBS or 10^8^ CFUs of pasteurized EcN (P_EcN_) at W3, 4 and 5. **(C–E)** EcNC101 colonization assessed by **(C)** PCR quantification of *clbP* gene level normalized to *16S* rRNA gene levels; and by CFUs in the **(D)** small intestine and **(E)** in the colon. **(F)** Body weight (mean ± SEM; between PBS and EcNC101 mice, repeated-measure ANOVA). **(G)** Tumor count in the small intestine. **(H)** Representative images of colonic tumors (left side: cecum, right side: rectum), scale bar = 1 cm. **(I)** Tumor size in the colon. **(J)** Colonic tumor grades (Chi-square Test). n.s. non-significant; *p < 0.05, **p < 0.01, ***p < 0.001, ****p < 0.0001. ANOVA, *N* = 9–14 per group.

Competition for space and nutrients in the gut is prevalent among bacterial species belonging to the same family as resources and niche requirements are similar (Hibbing et al., 2010). Since complete eradication of *pks + E. coli* from the gut microbiome is not possible (Raimondi et al., 2019), we tested whether limiting the growth of EcNC101 *via* competition decreased tumorigenesis by using a well-characterized probiotic, *E. coli* Nissle 1917 (EcN) (Scaldaferri et al., 2016) in co-cultures with EcNC101. As shown in Figure 4A, EcN was able to outcompete EcNC101 after 3 days of successive cycles of inoculations. Unfortunately, previous studies have shown that the probiotic properties of EcN cannot be dissociated from the production of colibactin (Olier et al., 2012), which may complicate the use of live EcN in the context of CRC. To circumvent the ability of EcN to produce colibactin, we tested pasteurized EcN and showed that *in vitro*, it significantly inhibited the growth of pathogenic EcNC101 as shown in Figure 4B.

We next tested pasteurized EcN in *Apc^Min/+^* mice. EcN101-colonized mice that received oral gavage with pasteurized EcN showed lower concentrations of colibactin in fecal samples as assessed by quantitative PCR (Figure 4C). Accordingly, CFUs of EcNC101 were lowered in the small intestine (Figure 4D) and 7-fold reduced in *Apc^Min/+^* mice gavaged with pasteurized EcN compared to mice receiving PBS (Figure 4E). As expected, EcNC101 colonization led to a decrease of body weight gain interdependent of further treatments (Figure 4F). In mice colonized with EcNC101, pasteurized EcN lowered tumor counts in the small intestine compared to mice treated with PBS (27 ± 3.0 vs. 38 ± 3.4 tumors; ±SEM Figure 4G) and was associated with smaller colonic tumors (1.53 ± 0.09 mm vs. 1.89 ± 0.13 mm; ±SEM Figures 4G–I). In addition, intramucosal carcinomas in the colon were only found in one out of 10 mice receiving pasteurized EcN whereas 6 out of 10 mice treated with PBS developed intramucosal carcinomas (Figure 4J).

Taken together, these results show that the deleterious effect of inulin in mice colonized by EcNC101 can be mitigated by using pasteurized EcN.

## 4. Discussion

Inulin is promoted as a dietary supplement and is used in processed foods to help increase the daily consumption of dietary fiber, which is habitually low in Western populations (Christoforou et al., 2021). Our study highlights a potential risk associated with inulin supplementation, especially in the presence of CRC-promoting bacteria such as *pks* + *E. coli* and raises questions about the safety of using inulin for individuals with specific gut microbiota compositions.

Colibactin-producing bacteria were detected in high percentage of the healthy population in a Japanese cohort (46%) (Shimpoh et al., 2017) and in a Canadian cohort (42%) (Oliero et al., 2022), whereas lower levels were found in other healthy cohorts: 20.8% in United States; 18.5% in Sweden; 7.1% in Iran and 4.3% in Malaysia (Arthur et al., 2012; Eklof et al., 2017; Iyadorai et al., 2020; Nouri et al., 2021). The reason for these differences are not clear at this point, but it might be caused by dietary and environmental factors, as well as vertical transfer from mother to infant (Tsunematsu et al., 2021) and horizontal transfer of the colibactin genes among bacteria (Putze et al., 2009).

### 4.1. The carcinogenic potential of colibactin-producing bacteria is enhanced by inulin supplementation

Our results confirms a previous study (Lopès et al., 2020) that demonstrates the carcinogenic potential of colibactin in mice fed a regular diet. We observed an increase in the number of tumors in the small intestine, and in the size and grade of colonic tumors in *Apc^Min/+^* mice colonized with the pathogenic strain EcNC101. Discrepancies with previous studies regarding enhanced tumor counts in the colon of EcN101 positive mice may be related to differences in *E. coli* strains employed and/or the duration of the studies (Bonnet et al., 2014; Lucas et al., 2020). Another colibactin-producing strain, *E. coli* MP13, was shown to promote carcinogenesis in the azoxymethane (AOM)/DSS CRC model (Zhu et al., 2019).

Inulin enhanced carcinogenesis in *Apc^Min/+^* mice colonized with *pks + E. coli*. Similarly, a diet supplemented with 10% inulin was found to increase the tumor promotion explained by higher level of cytosolic β-catenin (Pajari et al., 2003) and higher expression of cyclin D1 (Misikangas et al., 2008). In contrast, others have reported that dietary supplementation with 15% inulin decreased tumor sizes in syngeneic mice transplanted with melanoma cells subcutaneously (Li et al., 2020) and both 5 and 15% inulin supplemented diet reduced colonic tumoral load in AOM-treated *Apc^Min/+^* mice (Moen et al., 2016). In humans, inulin consumption was suggested to prevent CRC (Pool-Zobel and Sauer, 2007). However, more recent studies failed to find any association between inulin-type fructans and CRC prevention (Turati et al., 2022). Here we show that *pks + E. coli* negates the effect of inulin supplementation. When evaluating the effect of inulin, none of the previous studies assessed the presence of colibactin-producing bacteria, which could, at least partially, explain the variations between studies.

### 4.2. Inulin enhances colibactin-producing bacteria colonization in the gut

Increased carcinogenesis was associated with a greater abundance of EcNC101 in mice fed an inulin diet, further supporting previous studies showing that inulin promotes the growth of *pks + E. coli in vitro* (Oliero et al., 2021). Inulin fermentation supports the growth of many strains of *Bifidobacterium* and *Lactobacillus* (Zhu et al., 2020), including the probiotic *L. plantarum* 299 V, whereas *E. coli* strains are often compared as negative controls (Rossi et al., 2005; Jung et al., 2015). However, we found that the pathogenic strain EcNC101 may ferment inulin as a source for its growth, releasing monosaccharides into the culture medium.

### 4.3. Paraprobiotics can limit the expansion of colibactin-producing bacteria

We showed that the deleterious effect of EcNC101 increased-growth was mediated by the production of colibactin and was mitigated by addition of the probiotic EcN strain outcompeting pathogenic bacteria (Scaldaferri et al., 2016). EcN has been shown to be able to suppress the growth of *Salmonella typhimurium* by competing for iron acquisition through siderophore production (Deriu et al., 2013) and to limit the growth of related competitors, including pathobionts from the *Enterobacteriaceae* family, through the release of small antimicrobial molecules called microcins (Sassone-Corsi et al., 2016). This growth inhibition could be mediated by outer membrane vesicles (OMVs) produced by EcN that are able to stimulate the host immune responses (Cañas et al., 2016; Fábrega et al., 2016), to reinforce the epithelial barrier (Alvarez et al., 2016), and to reduce colitis in the DSS mouse model (Fábrega et al., 2017). OMVs from gram negative bacteria play a key role in nutrients intake, and in the release of bacteriocins (Schwechheimer and Kuehn, 2015) and proteases (Jan, 2017). Since EcN has been identified as potentially harmful for the host due to the presence of colibactin genes (Nougayrède et al., 2021), its pasteurization would have the advantage of resulting into colibactin degradation due to its high instability (Dougherty and Jobin, 2021). Here, we show that pasteurized EcN was able to suppress inulin-enhanced EcNC101 colonization in the gut of *Apc^Min/+^* mice resulting in lower tumorigenesis. Importantly, heat treatment has been shown to preserve the ability of EcN to inhibit bacterial competitive growth (Lagos, 2013). In addition, pasteurized EcN has been reported to have a direct anti-cancer effect through the regulation of signaling pathways in colonic cells (Alizadeh et al., 2020) and may lower inflammation in the intestine (Fábrega et al., 2017). The use of pasteurized EcN probiotic (paraprobiotic) instead of live bacteria has many advantages, the most important being the abolition of colibactin secretion, followed by greater safety, and improved logistic parameters of supplementation such as storage and shelf life (Piqué et al., 2019).

**Figure 5:**
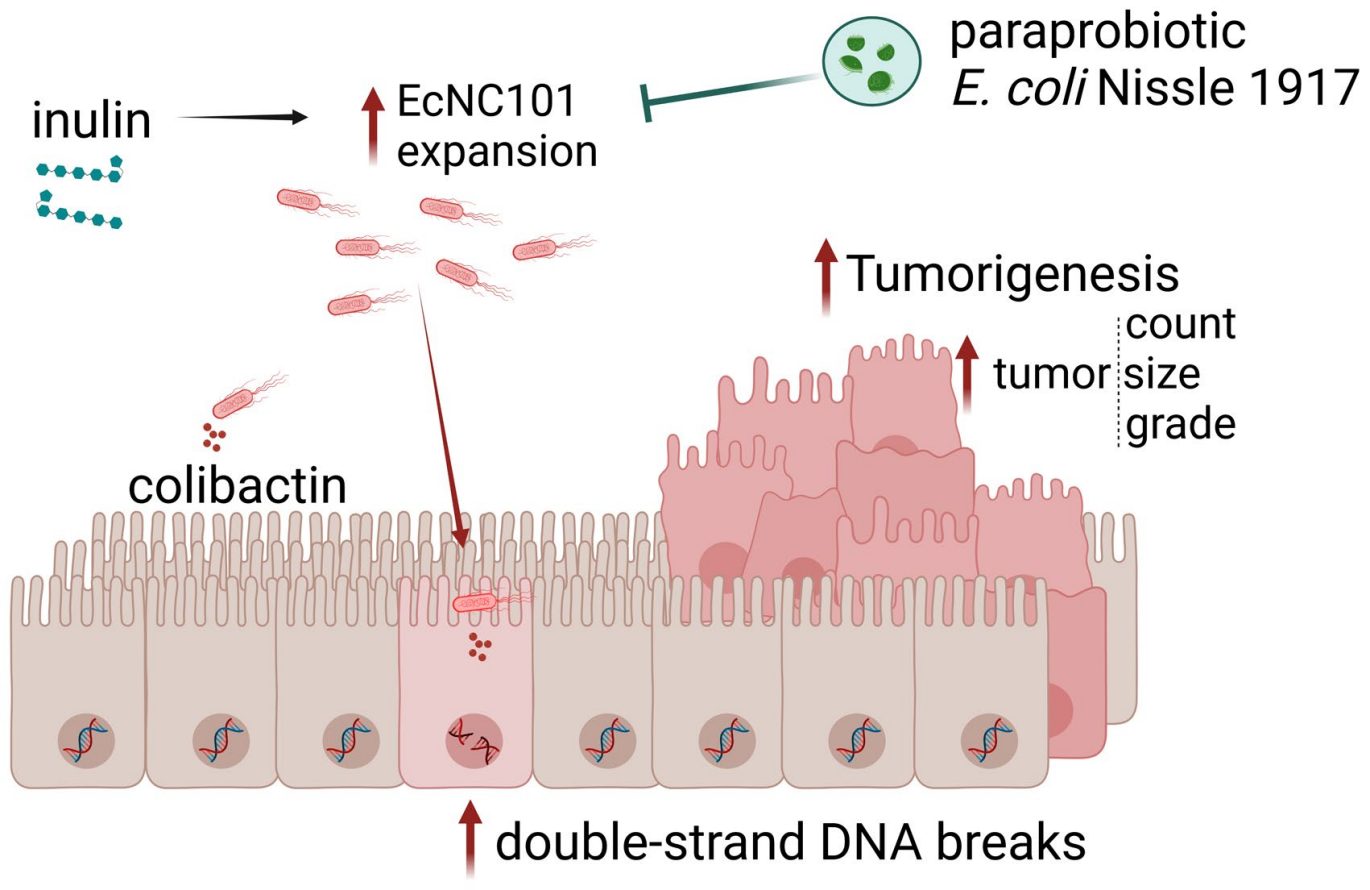
Schematic representation of the main findings of this work. Inulin supplementation promotes EcNC101 expansion in *Apc^Min/+^* mice, resulting in increased DSBs, cell proliferation, and tumorigenesis. The paraprobiotic EcN suppresses inulin-enhanced EcNC101 colonization in the gut of *Apc^Min/+^* mice resulting in lower tumorigenesis.

In conclusion, we show that inulin promotes EcNC101 growth in *Apc^Min/+^* mice, resulting in enhanced colonization, increased DSBs, cell proliferation, and tumorigenesis (Figure 5). These results suggest that inulin supplementation may not be appropriate for all individuals, depending on the composition of their gut microbiota. Our findings highlight the necessity of screening patients for *pks +* bacteria and providing them with appropriate preventive dietary counseling. Further studies are needed to investigate the interaction between dietary supplements and cancer-promoting bacteria, such as colibactin-producing bacteria.

## Data availability statement

The raw data supporting the conclusions of this article will be made available by the authors, without undue reservation.

## Ethics statement

The animal study was reviewed and approved by Institutional Animal Care Committee of the Centre de recherche du Centre Hospitalier de l’Université de Montréal (CRCHUM).

## Author contributions

The work reported in the paper has been performed by the authors, unless clearly specified in the text. MO contributed to the investigation. MO, RH, TC, GF, and AC contributed to the experiments. MO and MS contributed to conceptualization, validation, formal analysis, data visualization, and wrote and reviewed the original draft. MS contributed to the supervision of the study, resources, and funding acquisition. All authors have read and approved the version to be published.

## Funding

This work was supported by grants from the Canadian Institutes of Health Research [CIHR, grant PJT-159775] and the Natural Sciences and Engineering Research Council of Canada [NSERC, grant RGPIN-2018-06442] to MS. MO and TC are the recipients of the Canderel scholarship from the Institut du cancer de Montréal; and recipients of scholarships from the Université de Montréal, and RH received a scholarship from the Fonds de recherche du Québec-Santé [FRQ-S]/ Ministère de la Santé et des Services sociaux [MSSS; Resident Physician Health Research Career Training Program].

## Conflict of interest

The authors declare that the research was conducted in the absence of any commercial or financial relationships that could be construed as a potential conflict of interest.

## Publisher’s note

All claims expressed in this article are solely those of the authors and do not necessarily represent those of their affiliated organizations, or those of the publisher, the editors and the reviewers. Any product that may be evaluated in this article, or claim that may be made by its manufacturer, is not guaranteed or endorsed by the publisher.

## Footnotes

^1^ http://n2t.net/addgene:122138

^2^ http://n2t.net/addgene:78473

^3^ http://n2t.net/addgene:78468
