# Supplementary material for: Inulin impacts tumorigenesis promotion by colibactin-producing Escherichia coli in ApcMin/+ mice
Source: Front Microbiol. 2023 Feb 2;14:1067505. doi: 10.3389/fmicb.2023.1067505 (PMC9932902; doi:10.3389/fmicb.2023.1067505)
Supplement: Supplementary file 1 [file Data_Sheet_1.docx]

Supplementary Material

# Supplementary Figures and Tables

## Supplementary Table

**Supplementary Table 1. Primers**

| **Name** | **Sequence** | **Reference** |
| --- | --- | --- |
| Ecol16S-Fw | GTTAATTTTGCTCATTGA | (Gao et al., 2011) |
| Ecol16S-Rv | ACCAGGGTATCTAATCCTGTT | (Gao et al., 2011) |
| 16S-Fw | CGGCAACGAGCGCACCCC | (Lane et al., 1985) |
| 16S-Rv | CCATTGTAGCACGTGTGTAGC | (Lane et al., 1985) |
| clbP-Fw | GATGTGGCTAGTCAGAAAGC | (Homburg et al., 2007) |
| clbP-Rv | CATAATTGGCGGAGGCATAG | (Homburg et al., 2007) |
| clbA-Fw | CTCCACAGGAAGCTACTAAC | (Homburg et al., 2007) |
| clbA-Rv | CGTGGTGATAAAGTTGGGAC | (Homburg et al., 2007) |
| CheckClbp_Fw | TATTGTCCGACAGCACGCTA | This study |
| CheckClbP_Rv | GTATGGGGTCCATCGAGCG | This study |
| CheckΔClbP_Rv | GTTTACCGGAAATGCCAGCC | This study |
| Plantarum_Fw | GCTGGCAATGCCATCGTGCT | (Kim et al., 2020) |
| Plantarum_Rv | TCTCAACGGTTGCTGTATCG | (Kim et al., 2020) |

GAO, W., ZHANG, W. & MELDRUM, D. R. 2011. RT-qPCR based quantitative analysis of gene expression in single bacterial cells. *J Microbiol Methods,* 85**,** 221-7.

HOMBURG, S., OSWALD, E., HACKER, J. & DOBRINDT, U. 2007. Expression analysis of the colibactin gene cluster coding for a novel polyketide in Escherichia coli. *FEMS Microbiol Lett,* 275**,** 255-62.

KIM, E., YANG, S. M., LIM, B., PARK, S. H., RACKERBY, B. & KIM, H. Y. 2020. Design of PCR assays to specifically detect and identify 37 Lactobacillus species in a single 96 well plate. *BMC Microbiol,* 20**,** 96.

LANE, D. J., PACE, B., OLSEN, G. J., STAHL, D. A., SOGIN, M. L. & PACE, N. R. 1985. Rapid determination of 16S ribosomal RNA sequences for phylogenetic analyses. *Proceedings of the National Academy of Sciences of the United States of America,* 82**,** 6955-6959.

## Supplementary Figures


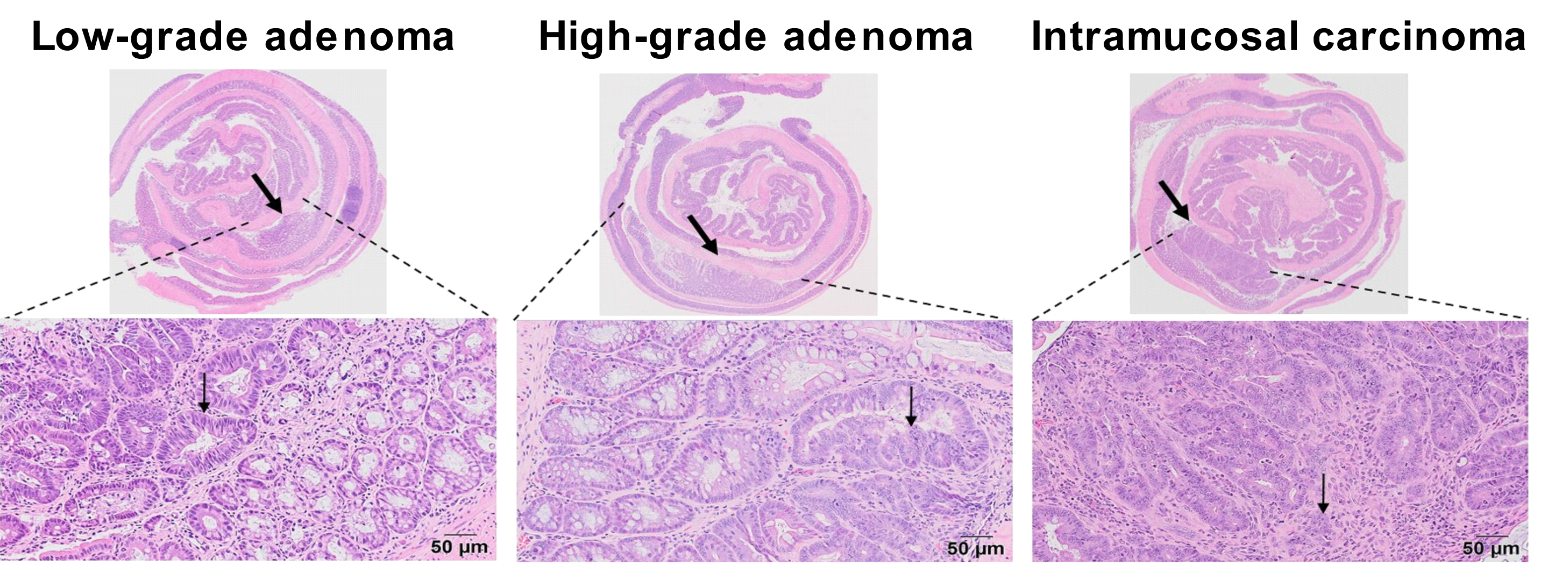


**Supplementary Figure 1.** **Tumor grading in the colon of *Apc^Min/+^* mice.** (H&E staining). Low-grade adenoma: hyperplasic cells; high-grade adenoma: apical nucleus migration; intramucosal carcinoma: dysplastic cells migration in the mucosa (main image, 40× magnification; inset image; scale bar = 50 µm).

**
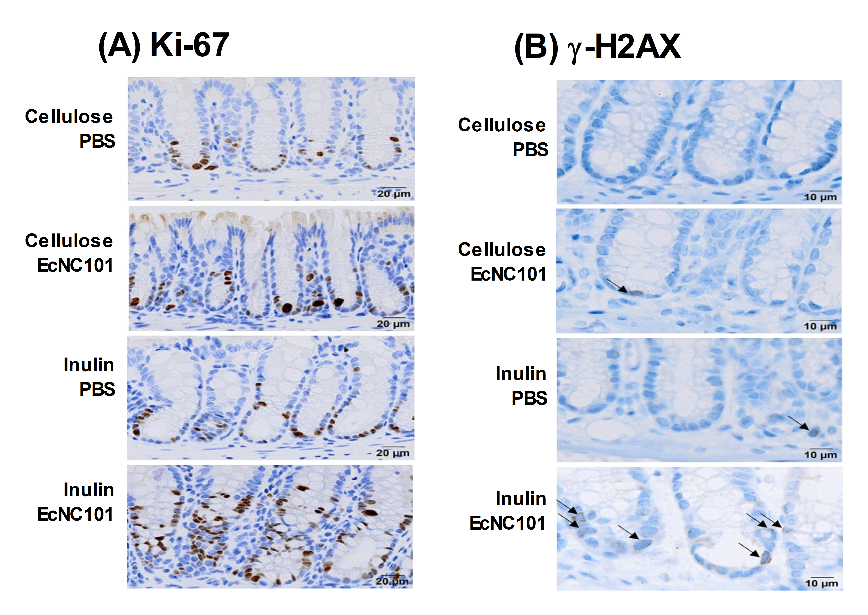
**

**Supplementary Figure 2.** **Tumor grading in the colon of *Apc^Min/+^* mice.** (**A**) Cell proliferation assessed by Ki-67 immunohistochemistry (scale bar = 20 µm). (**B**) DSBs assessed by γ-H2AX immunohistochemistry (black arrows indicate phosphorylated-H2AX positive cells; scale bar = 10 µm).
